# Selectivity control in Pt-catalyzed cinnamaldehyde hydrogenation

**DOI:** 10.1038/srep09425

**Published:** 2015-03-24

**Authors:** Lee J. Durndell, Christopher M. A. Parlett, Nicole S. Hondow, Mark A. Isaacs, Karen Wilson, Adam F. Lee

**Affiliations:** 1 European Bioenergy Research Institute, Aston University, Birmingham B4 7ET, UK; 2 Institute for Materials Research, University of Leeds, Leeds LS2 9JT, UK

## Abstract

Chemoselectivity is a cornerstone of catalysis, permitting the targeted modification of specific functional groups within complex starting materials. Here we elucidate key structural and electronic factors controlling the liquid phase hydrogenation of cinnamaldehyde and related benzylic aldehydes over Pt nanoparticles. Mechanistic insight from kinetic mapping reveals cinnamaldehyde hydrogenation is structure-insensitive over metallic platinum, proceeding with a common Turnover Frequency independent of precursor, particle size or support architecture. In contrast, selectivity to the desired cinnamyl alcohol product is highly structure sensitive, with large nanoparticles and high hydrogen pressures favoring C = O over C = C hydrogenation, attributed to molecular surface crowding and suppression of sterically-demanding adsorption modes. In situ vibrational spectroscopies highlight the role of support polarity in enhancing C = O hydrogenation (through cinnamaldehyde reorientation), a general phenomenon extending to alkyl-substituted benzaldehydes. Tuning nanoparticle size and support polarity affords a flexible means to control the chemoselective hydrogenation of aromatic aldehydes.

Chemoselectivity is a cornerstone of catalysis, permitting the targeted modification of specific functional groups within complex starting materials^1^,^2^,^3^,^4^,^5^. This ability to activate and transform only certain chemical functionalities without the use of protecting groups, and attendant improvements in atom efficiency (and waste minimisation), also underpins catalysis' green credentials^6^,^7^,^8^. Catalytic hydrogenation of organic compounds possessing multiple unsaturated bonds such as α,β-unsaturated aldehydes is particularly challenging^9^,^10^,^11^,^12^, necessitating active sites able to discriminate closely related moieties, and in some instances achieve preferential activation of a more thermodynamically stable function. Platinum is widely employed in heterogeneous catalytic hydrogenation, able to reduce a plethora of functional groups, including C = C^13^, C≡C^14^, C = O^15^, C≡N^16^, NO_2_^17^ and aromatics^18^ with molecular hydrogen. Selective hydrogenation of allylic and benzylic aldehydes to unsaturated alcohols is a commercially important industrial process widely utilised within the flavor and fragrance, agrochemical, and pharmaceutical sectors^10^,^19^, however the development of requisite heterogeneous catalysts has been hindered by the thermodynamic stability of C = O relative to C = C bonds and lack of insight into fundamental structure-function relations^10^.

Tsang and co-workers attempted to elucidate the roles of geometric and electronic effects in Pt catalyzed cinnamaldehyde (CinnALD) hydrogenation to cinnamyl alcohol (CinnOH) through studies of oleic acid/oleylamine stabilised mono- and bimetallic colloidal Pt nanoparticles. Selectivity towards CinnOH exhibited a strong dependence on Pt nanoparticle size, with low coordination sites favoring C = C hydrogenation^20^,^21^. In contrast, Zhu and Zaera found that CinnOH selectivity was insensitive to the size of silica supported Pt nanoparticles^22^, although rates of CinnALD hydrogenation were structure sensitive, with (111) facets prevalent on larger particles accounting for a five-fold increase in Turnover Frequency (TOF) between 1.3 and 2.4 nm particles. Bimetallic catalysts formed via either one-pot synthesis or doping of Pt nanoparticles^20^,^21^,^23^,^24^, can afford enhanced selectivity to CinnOH, although decoupling the role of promoters in blocking unselective sites versus electronic modification of platinum itself has not proved possible. Surface science studies of crotonaldehyde, an aliphatic analogue of CinnALD, have demonstrated that the molecular adsorption geometry is critical in directing final product selectivity over Pt(111) surfaces, with high coverages lifting the C = C bond and tilting the C = O bond with respect to the surface^25^,^26^. In an extension of this concept, thiolate adsorption onto Pt surfaces^27^ has been employed to achieve selective C = O activation^28^, with phenylated thiols facilitating tunable, specific non-covalent interactions with CinnALD and consequent molecular orientation with respect to the surface of Pt/Al_2_O_3 _catalysts thereby boosting CinnOH selectivity by 70%. This promotion was attributed to π-stacking interactions between self-assembled monolayers of such thiols and the phenyl ring of CinnALD, which did not compromise the rate of product formation^28^. Guo et al. have shown that confinement of Pt nanoclusters within the cavity of metal-organic frameworks also promotes CinnOH selectivity; steric constraints on the CinnALD geometry is believed to hinder C = C planar adsorption, again promoting C = O activation^29^. Despite this progress, kinetics of the CinnALD hydrogenation reaction network have not yet been mapped in detail over any heterogeneous catalyst, while for platinum there has been no systematic study on the impact of particle size (over a wide range) or H_2_ pressure, or of support properties which influence not only CinnALD hydrogenation^30^ but also crotonaldehyde^31^ and citral hydrogenation^32^,^33^. Consequently the nature of the active site remains a matter for speculation, and little is known regarding the effect of substituents, or the extent to which mechanistic models can be extended to other conjugated aldehydes.

Here, we resolve the preceding controversies, elucidating the reaction mechanism and kinetic pathways for CinnALD selective hydrogenation over two families of silica supported catalysts of tailored hydrophilicity and Pt nanoparticle size. To this end, the bulk and surface properties of nanoparticles and silica supports were characterised by XPS, XRD, HRTEM, SEM, CO chemisorption, DRIFTS, ATR-IR and porosimetry. Kinetic profiling revealed that CinnALD hydrogenation was structure insensitive, proceeding equally well over small (<2 nm) or large (~15 nm) particles, however C = O hydrogenation was profoundly structure sensitive, requiring large metal ensembles. Complementary in situ powder XRD and operando ATR-IR measurements provided valuable insight into the respective roles of hydrogen pressure and support functionality in regulating C = O versus C = C hydrogenation, with the resulting insight successfully predicting the behaviour of α-methyl-trans-cinnamaldehyde and benzylic aldehyde hydrogenation over Pt catalysts, highlighting the generality of the concepts identified.

## Results and Discussion

### Catalyst characterization

Successful genesis of a hexagonal close packed *p6mm* pore architecture within the parent mesoporous silica support (characteristic of SBA-15) was confirmed by low angle powder X-ray diffraction (Figure S1). Nitrogen porosimetry also demonstrated type IV isotherms with type H1 hysteresis as expected for SBA-15, with a BET surface area, mean BJH pore size and narrow mesopore size distributions consistent with literature values (Table S1)^34^,^35^. The fumed silica exhibited a type II isotherm, indicating a non-porous or macroporous material, and low BET surface area. HRTEM shown in Figure S2 confirmed the ordered mesopore network of SBA-15. Complimentary measurements on the two Pt-impregnated silica families evidenced pore arrangements and mesopore diameters comparable to those of the parent SBA-15 and fumed silica (Table S2). However, BET surface areas decreased for both supports with increasing Pt loading, with the Pt/SBA-15 materials exhibiting the greatest loss (up to 20%), which we attribute to micropore blockage consistent with t-plot analysis in Table S2. Such surface area losses are in quantitative agreement with those reported following Pd impregnation of the same supports^35^. The smaller loss in surface area for the Pt/SiO_2_ materials was consistent with deposition of platinum nanoparticles predominantly over the (proportionately larger) external surface area of the fumed support.

Wide angle powder XRD (Figure S4) revealed exclusively fcc platinum metal over both silicas. Platinum nanoparticle sizes estimated from these reflections increased with loading, from 5.4 nm (0.5 wt%) to 15 nm (2 wt%) for Pt/SBA-15, and 8.4 to 17 nm for the analogous Pt/silicas; larger crystallites are expected for the latter due to the lower surface area of the fumed silica. The size, dispersion and oxidation state (Table S2, Figure S6) of silica supported platinum nanoparticles were sensitive to metal loading. Nanoparticle diameter increased linearly with Pt loading over both silicas (Figure S6a), while platinum dispersion (surface Pt^0^ content) decreased (increased) monotonically with diameter between 2 and 8 nm before reaching a plateau for larger sizes (Figure S6b). Platinum dispersion and surface oxidation state were solely a function of nanoparticle size for both fumed and mesoporous silicas, irrespective of whether nitrate or chloride precursor were employed, consistent with the generally-held view of silica as a weakly interacting support; no unusual Pt redispersion was observed, as has been postulated via Pt(IV)Cl_x_ surface complexes when using a hexachloroplatinic acid precursor^36^.

### Cinnamaldehyde hydrogenation

**Figure 1:**
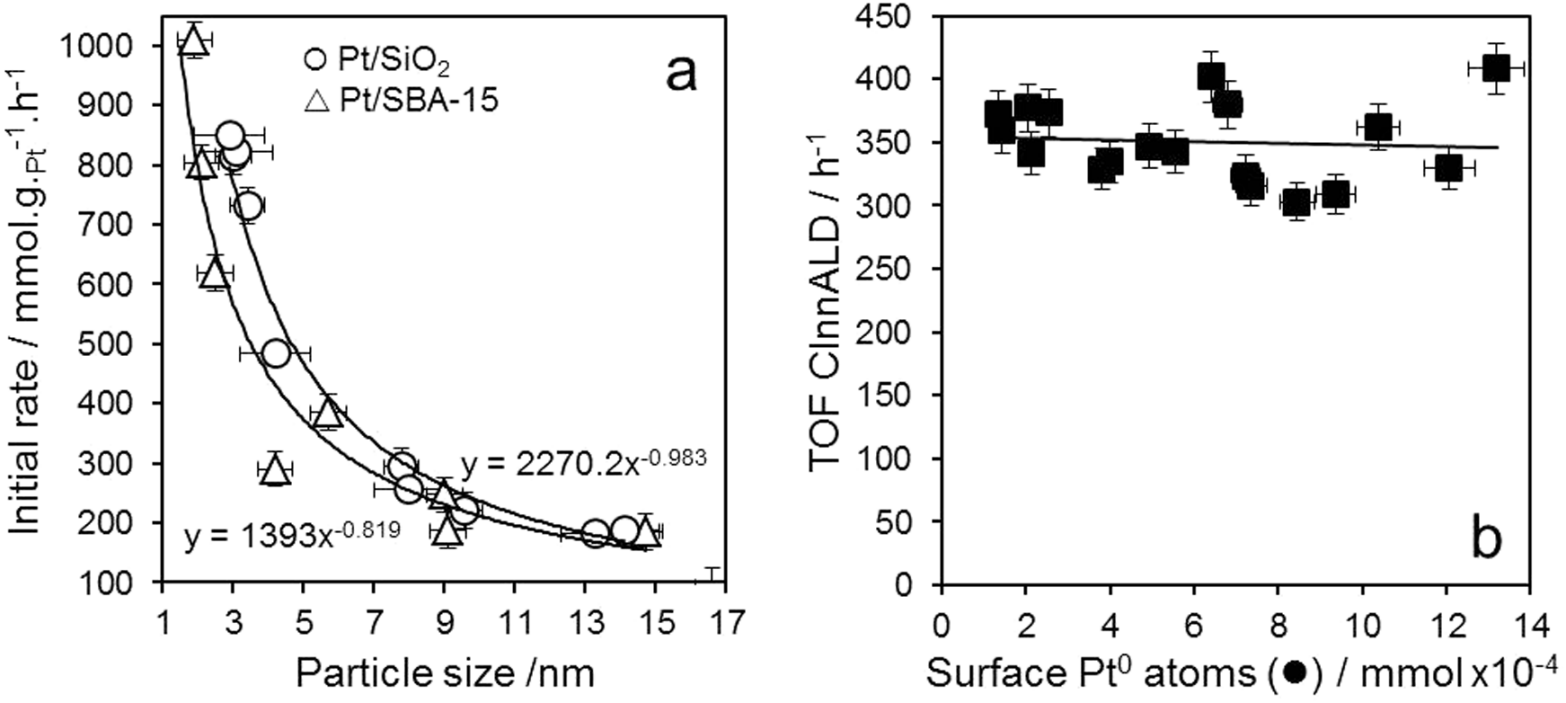
(a) Initial rate of CinnALD hydrogenation over silica supported platinum catalysts at 1 bar as a function of particle size; and (b) corresponding turnover frequencies for CinnALD hydrogenation.

The selective hydrogenation of CinnALD was subsequently studied over both Pt/SiO_2_ and Pt/SBA-15 catalyst series (Figure S7-14). Initial hydrogenation rates fell dramatically with increasing nanoparticle size (Figure 1a), exhibiting an inverse proportionality with particle diameter, precisely as would be anticipated if reactivity was dictated solely by the geometric platinum surface area, irrespective of the local coordination number of surface atoms or electronic structure. This structure insensitivity is confirmed by calculating the corresponding TOFs for CinnALD hydrogenation (Figure 1b), derived by normalizing the initial rate to the surface density of metallic Pt atoms determined via CO chemisorption and XPS, which were particle size (and support and precursor) invariant at around 350 h^−1^ under 1 bar H_2_ for all catalysts. Apparent activation energies for the highest loading Pt/SiO_2 _and Pt/SBA-15 catalysts were also identical at 21 kJ.mol^−1^, implicating a common reaction mechanism for CinnALD hydrogenation over both supports. Similar TOFs between 200 and 350 h^−1^ have been reported for a narrow size distribution of platinum and ruthenium nanoparticles supported on carbon nanofibers in atmospheric pressure CinnALD hydrogenation^37^. However, this finding stands in contrast to a recent report of room temperature CinnALD hydrogenation at 10 bar H_2_ over Pt/Aerosil silica catalysts, for which the TOF increased from 396 to 1836 h^−1^ with increasing Pt particle size over the narrow range from 1.3 to 2.4 nm^22^; albeit, this previous study noted significant errors in both activity and nanoparticle size, relying upon indirect estimates of the surface Pt atom density via TEM from which to determine TOFs. Considering the systematic behavior of the 18 different Pt/silica catalysts in the present work, *we find no evidence that CinnALD hydrogenation is favoured over flat surfaces present on larger platinum nanoparticles*. The inverse proportionality of CinnALD hydrogenation initial rate on particle size is consistent with a direct correlation between activity and the geometric surface Pt atom density, i.e. the rate of cinnamaldehyde hydrogenation depended solely on the surface area:volume ratio (∝ particle diameter^−1^), with no preference for specific platinum facets. The latter also suggests that all catalysts underwent rapid in situ reduction to present a similar, metallic platinum surface species exhibiting similar electronic structure.

**Figure 2:**
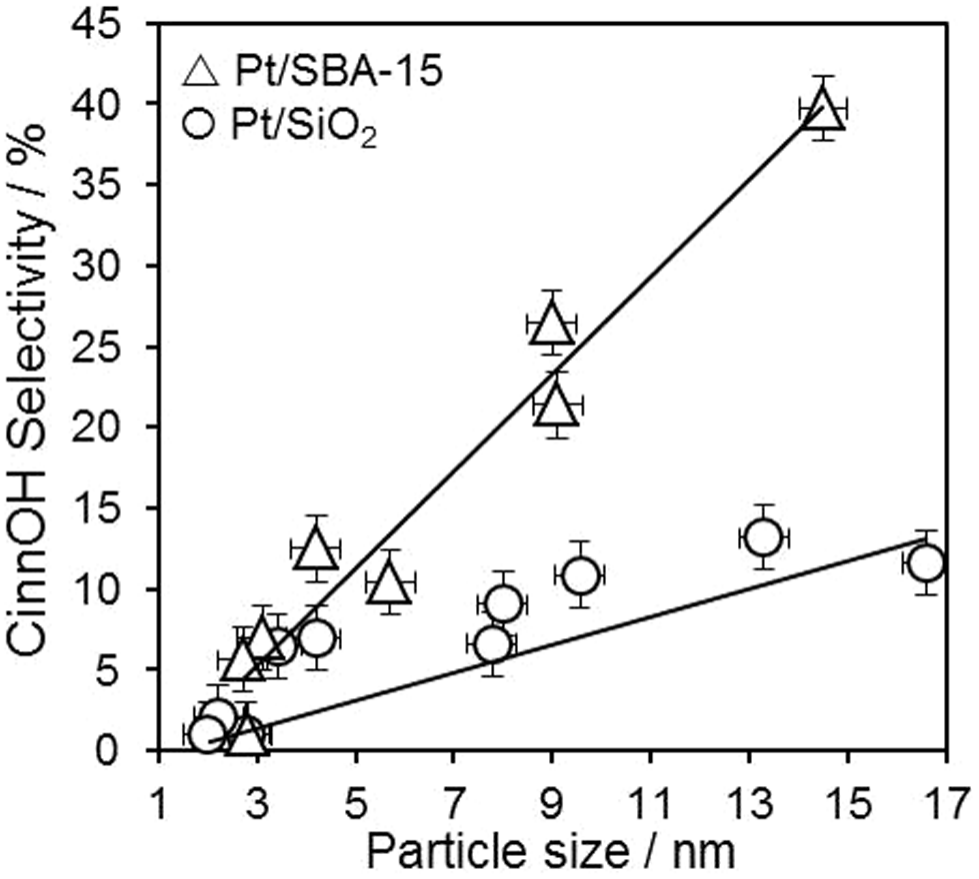
CinnOH selectivity after 7 h CinnALD hydrogenation over silica supported platinum catalysts at 1 bar as a function of particle size.

By comparison, selectivity towards the desired CinnOH product was strongly dependent on particle size and support morphology (Figure 2), increasing linearly from essentially zero over the smallest (2 nm) particles, to 12% and 40% for 15 nm particles on the fumed silica and mesoporous SBA-15 supports respectively. Identical trends were observed during the first hour of reaction (Figure S12). This particle size dependence is consistent with a number of previous reports for Ru^38^,^39^, Co^40^ and Pt^20^,^37^,^41^ catalysts, and is generally attributed to an increase in the density of Pt (111) facets relative to lower coordination sites over larger nanoparticles, which hamper close approach of the C = C bond and hence favour C = O hydrogenation^20^,^21^,^42^,^43^. This hypothesis is supported by extended Hückel calculations of Delbecq and Sautet which revealed that di-σ_CC_ CinnALD adsorption is strongly destabilised over Pt(111) facets with respect to a di-σ_CO_ mode, and hence favour C = O hydrogenation, in comparison with Pt(100) and stepped facets which stabilise a co-planar η_4_ di-σ_CO_ + π_C = C_ or trihapto π_C = C_ + (O) mode, and hence favour C = C hydrogenation^44^. Recent DFT calculations indicate that the activation barrier to C = C hydrogenation of allylic aldehydes and ketones over Pt(111) remains lower than that of C = O hydrogenation, but also suggest that phenyl substitution *α* to the C = C bond should slow its rate of hydrogenation^45^. This body of literature stands in contrast to the high pressure study of Zhu and Zaera^22^, who reported a similar low initial selectivity (~20%) towards CinnOH over Pt/Aerosil silica to that in the present work, but surprisingly found this selectivity insensitive to particle size or CinnALD conversion (below 80%) for sub 2.4 nm nanoparticles.

Other major reaction products were 3-phenyl propionaldehyde > 3-phenyl propan-1-ol > ethylbenzene: high selectivity to the saturated aldehyde demonstrates that undesired C = C hydrogenation competes strongly with C = O hydrogenation, with ethylbenzene a by-product of 3-phenyl propionaldehyde and/or 3-phenyl propan-1-ol hydrogenolysis. CinnOH selectivity increased continuously over the course of reaction for all catalysts, doubling its value between 1 and 7 h. This improved selectivity occurred at the expense of 3-phenyl propionaldehyde and 3-phenyl propan-1-ol for the Pt/SBA-15 and Pt/SiO_2_ catalysts respectively (Figure S9). Note that the principal product during the early stage of reaction over 2 wt% Pt/SiO_2_ was 3-phenyl propan-1-ol, a secondary product arising from hydrogenation of either cinnamyl alcohol or 3-phenyl propionaldehyde. The absence of CinnOH primary product can be rationalised by considering that the rate of its hydrogenation (and hence removal from the reaction mixture) is >40 times faster than its initial rate of formation (see below), accounting for a very low CinnOH and high concentration of 3-phenyl propan-1-ol secondary product; onstream deactivation of this rapid CinnOH hydrogenation step would account for its subsequent accumulation at higher conversions. Literature in this regard is conflicted, with a strong product dependence on CinnALD conversion reported over Pt^46^, Ir^47^ and Ru^39^, while other researchers note minimal change in CinnOH selectivity for conversions <80%^20^,^37^,^48^,^49^,^50^. From the present kinetic investigation we can conclude that C = C hydrogenation is initially heavily favoured over Pt/SBA-15, but that this pathway is rapidly switched off during the early stages of CinnALD hydrogenation, possibly due to surface crowding by strongly bound adsorbates^49^.

The observed structure sensitivity of CinnOH selectivity upon Pt nanoparticle size seen in Figure 2 was five times greater for Pt/SBA-15 catalysts. Since CinnALD hydrogenation proceeds with a common TOF over both supports, this difference cannot be readily ascribed to differential mass-transport (which should in any event favour more rapid removal of the reactively-formed CinnOH for Pt/SiO_2_, wherein reaction occurs largely on the external surface area, and hence higher selectivity to this desired product). Net CinnOH selectivity is a function of both the rate of C = O (versus C = C) hydrogenation of the CinnALD reactant, and of secondary hydrogenation of the allylic alcohol product to 3-phenyl propanol. The preceding observations can only therefore be understood by also considering the support dependence of CinnOH hydrogenation; we therefore undertook parallel studies employing CinnOH and 3-phenyl propionaldehyde as substrates.

**Figure 3:**
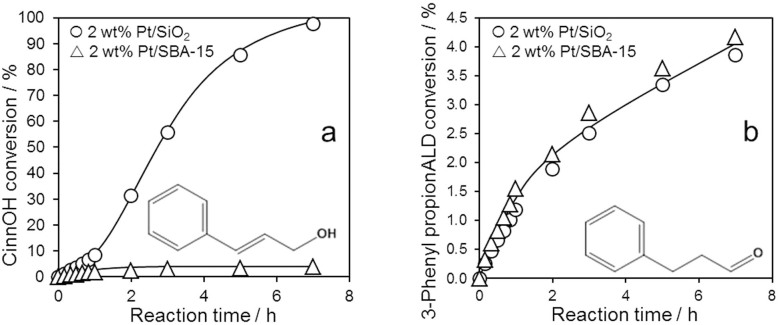
(a) CinnOH and (b) 3-phenyl propionaldehyde hydrogenation over 2 wt% silica supported platinum catalysts at 1 bar.

Figure 3a highlights a dramatic difference in CinnOH reactivity over fumed silica versus SBA-15 supported Pt nanoparticles of similar loading (~2 wt%) and size (~15 nm), in precisely the regime wherein the most significant differences in CinnALD hydrogenation to the alcohol were identified in Figure 2. However, the question remains as to whether the support also influences the rate of *CinnOH formation*. Selective hydrogenation studies of 3-phenyl propionaldehyde, the primary product resulting from CinnALD C = C hydrogenation, revealed identical rates of its removal over both 2 wt% catalysts (Figure 3b), and hence selectivity to this saturated aldehyde is determined only by the relative rates of CinnALD C = C versus C = O hydrogenation. Since CinnALD selectivity towards 3-phenyl propionaldehyde differs at 39% (SBA-15) versus 51% (SiO_2_), we can conclude that the support does somewhat impact upon the rates of C = C versus C = O hydrogenation, and hence also influences the rate of CinnOH formation. In other words, the observed differences in selective hydrogenation of CinnALD to CinnOH over the fumed versus mesoporous supports are dominated by their differing reactivity towards the unsaturated alcohol primary product, but are also influenced by their interaction with CinnALD. The former finding is in excellent agreement with DFT calculations by Laref and co-workers for allylic aldehyde hydrogenation over Pt(111) model surfaces, which showed that selectivity to unsaturated alcohols is determined predominantly by the strength of their binding^51^.

**Figure 4:**
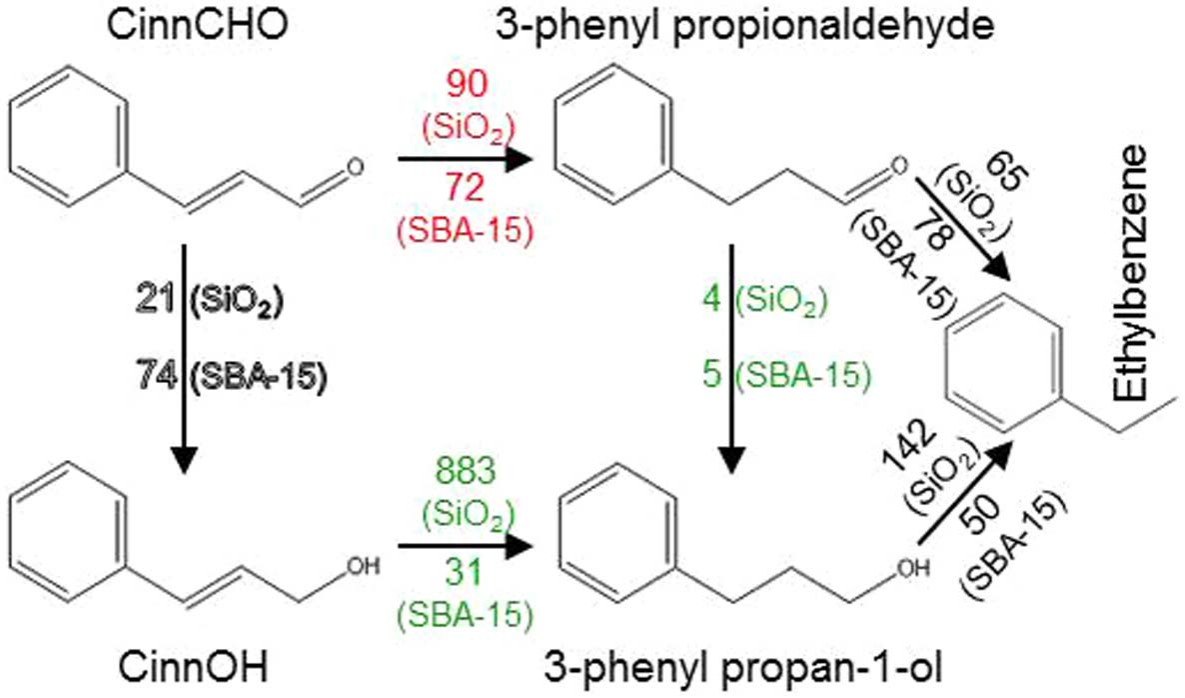
Kinetic network for CinnALD hydrogenation over 2 wt% Pt/silica catalysts. Values refer to the initial rates of each step in mmol.h^−1^.g_Pt_^−1^.

A complete reaction network for CinnALD hydrogenation is shown in Figure 4 below, with reaction rates determined for each step over the highest loading Pt catalysts. This highlights the critical reactivity difference between the two silica supports, namely that fumed silica favor C = C hydrogenation of both CinnALD and the desired CinnOH product, whereas SBA-15 is more selective towards CinnOH formation and suppresses its subsequent removal.

**Figure 5:**
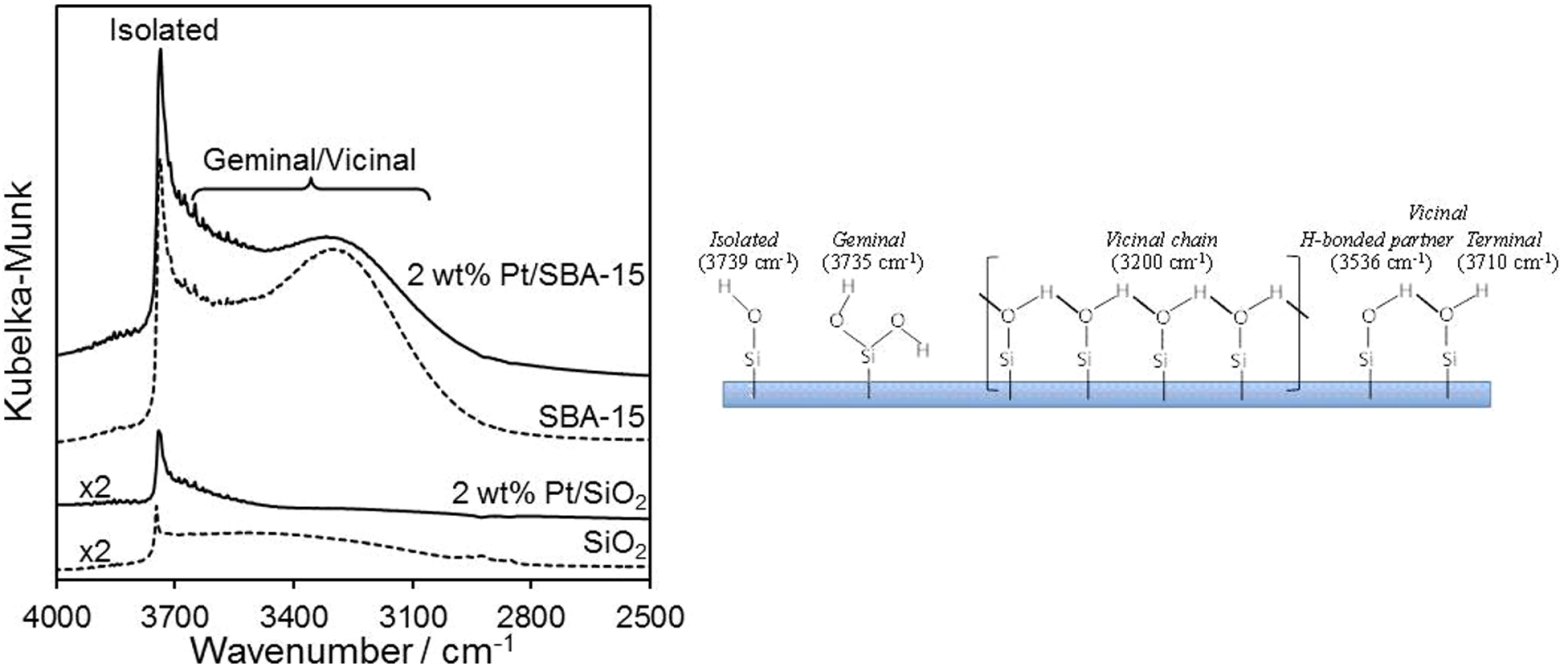
In vacuo DRIFT spectra of 2 wt% silica supported Pt catalysts dried at 200°C; assignment of silanol functions and associated vibrational frequencies for silica surfaces.

Support effects upon CinnALD hydrogenation and CinnOH selectivity have been noted for carbon^30^,^50^ and oxide^46^,^52^ supported Pt NPs. In the former case, annealed carbon nanofibers were postulated to produce non-polar surfaces favouring CinnALD adsorption via the benzene ring directly on the support; however, such non-polar nanofibers were far less selective to CinnOH than their oxygen-rich, acidic counterparts. In contrast, Ji et al. recently reported graphene-based catalysts as more selective towards CinnOH than Vulcan carbon analogues^30^ which possess more polar surfaces^53^, attributed to the higher proportion of Pt metal present on graphene. Lewis acidic Al-SBA-15 and Al_2_O_3_^46^ supports also exhibit enhanced CinnOH selectivity, hypothesised due to preferential adsorption of the polar C = O function at sites adjacent to Pt nanoparticles. In order to identify whether the differing reactivity of our fumed silica and SBA-15 supported platinum catalysts seen in Figure 2, 3 was likewise a consequence of surface polarity, DRIFT spectra of the parent supports and high loading Pt catalysts were compared. Figure 5 highlights a striking difference in the silanol surface density and coordination mode between the two silica supports: mesoporous SBA-15 possesses almost twice the density of surface silanols of the fumed silica (3.0 vs. 1.6 mmol.g^−1^ respectively), and is dominated by geminal/vicinal silanol groups whereas fumed silica only exhibits isolated silanols. Vicinal silanols comprise extended, hydrogen bonded hydrophilic patches^54^, and hence our SBA-15 catalysts are indeed extremely polar compared to those prepared from fumed silica (whose hydrophobicity as a consequence of isolated silanols has been recently described^55^). Since the physicochemical properties of Pt nanoparticles in terms of electronic charge (XPS), phase (XRD) and size (TEM/CO chemisorption) are essentially identical over both supports, it therefore seems entirely plausible that the higher selectivity to CinnOH of the Pt/SBA-15 arises from molecular re-orientation of the CinnALD reactant and/or reactively-formed hydrogenation products. Hence SBA-15 is expected to *disfavour* CinnALD and CinnOH adsorption geometries over platinum which require close approach of the apolar phenyl ring to the support surface, as necessary to effect C = C hydrogenation at the nanoparticle-support perimeter, and conversely *favour* adsorption configurations in which the C = O function is proximate to the support with the molecular plane oriented away from the surface. A similar concept has been advanced for crotonaldehyde (the aliphatic C_4_ analogue of CinnALD) over a Pt(111) single crystal wherein molecular tilting distances the C = C bond from the surface while activating the C = O bond towards hydrogenation^28^.

**Figure 6:**
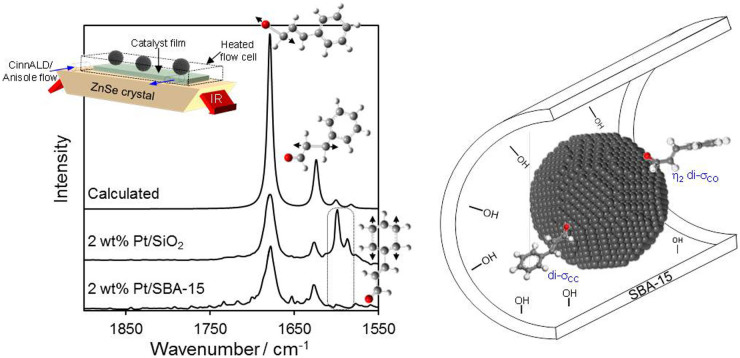
(left) In situ ATR-IR spectra of 2 wt% silica supported Pt catalysts films under a flowing CinnALD/anisole solution at 90°C and (right) illustration of unfavorable aromatic-surface interaction arising from adoption of di-σ_CC_ versus di-σ_CO_ CinnALD adsorption on platinum nanoparticles within polar SBA-15 pores.

The preceding hypothesis was tested via an in situ ATR-IR study of CinnALD adsorption from a 0.84 M anisole solution over films of 2 wt% Pt/SiO_2_ and 2 wt% Pt/SBA-15 catalysts at 90°C. This mimics the actual reaction conditions utilised during our catalytic studies of CinnALD hydrogenation, but without the presence of dissolved hydrogen and attendant complications arising from IR signatures due to hydrogenation products. Vibrational spectra over both catalyst films were temperature independent between room temperature and 90°C, and exhibited characteristic ν_C = O_ and symmetric ν_C = C_ bands of the parent CinnALD at 1678 and 1624 cm^−1^ respectively (Figure 6). However, a key difference is apparent in the aromatic C = C regime, wherein bands at 1600 and 1594 cm^−1^ associated with the aromatic C = C stretches are absent from Pt nanoparticles dispersed over the polar SBA-15 support, indicating loss of conjugation across CinnALD due to adsorption through the carbonyl function and associated molecular reorientation relative to that adopted on nanoparticles residing on the less polar fumed silica, consistent with the above model. CinnALD hydrogenation proceeds with a common apparent activation energy of 21 kJ.mol^−1^ over 2 wt% SBA-15 and fumed silica, supporting the notion that the differing selectivity of these catalysts reflects different modes of CinnALD adsorption over each support, rather than e.g. types of hydrogenation active sites. In summary, the higher selectivity of Pt/SBA-15 towards CinnOH during CinnALD hydrogenation appears associated with a molecular reorientation of the phenyl ring due to repulsive interactions with surface silanols, facilitating preferential di-σ_CO_ adsorption and subsequent C = O hydrogenation (illustrated in Figure 6).

**Figure 7:**
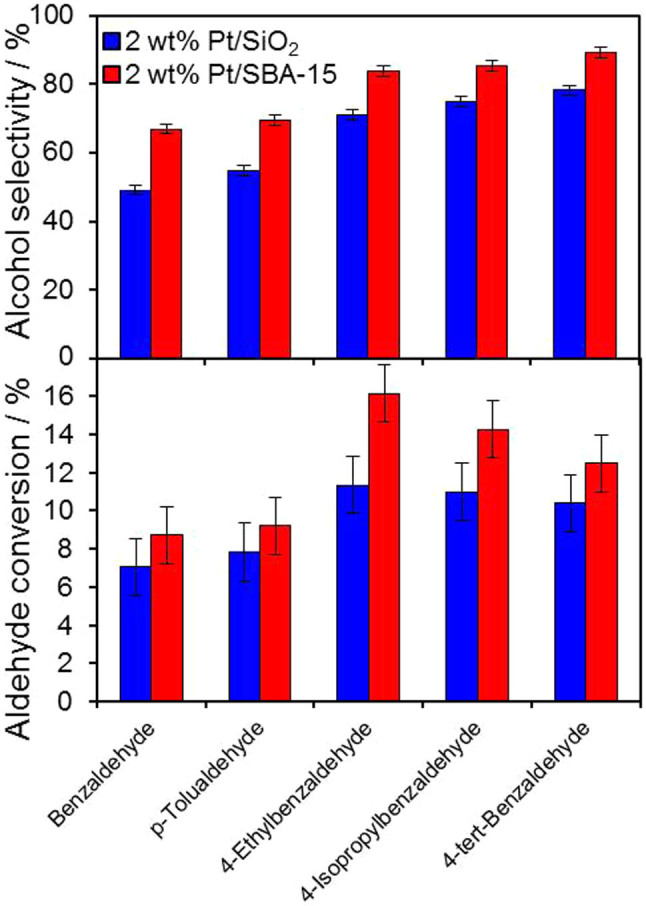
Performance of 2 wt% silica supported Pt catalysts at 7 h for the hydrogenation of substituted benzaldehydes under standard reaction conditions employed for CinnALD hydrogenation.

Our proposition that polar supports favour selective C = O hydrogenation of aromatic aldehydes (through molecular reorientation) was tested for the hydrogenation of benzylic aldehydes over the same 2 wt% Pt/SBA-15 and Pt/SiO_2_ catalysts. Figure 7 summarises the resulting performance, from which it is evident that the more polar Pt/SBA-15 outperforms the fumed silica support in respect of C = O versus C = C hydrogenation/hydrogenolysis for all substrates, as postulated. It has been speculated that methyl substituents stabilise adsorbed π-complexed aromatics resulting in higher barriers to ring hydrogenation^56^,^57^, rationalising the superior selectivity we observe towards alkyl-substituted benzylic alcohols versus benzyl alcohol. Surprisingly, Pt/SBA-15 was also *more active* towards all five benzylic aldehydes than Pt/SiO_2_, whereas it was marginally *less active* towards CinnALD (13% versus 19% conversion respectively). This may be a consequence of faster desorption of the less polar benzylic products away from the SBA-15 surface. Figure 7 shows that electron-donating alkyl substituents accelerated benzaldehyde hydrogenation over both silica supports, presumably via activation of the carbonyl function (in addition to the aromatic ring).

Liquid phase catalytic hydrogenations typically exhibit strong positive reaction orders in hydrogen partial pressure, reflecting the increased availability of atomic hydrogen as a consequence of higher solubility (Henry's Law). The impact of hydrogen pressure upon CinnALD hydrogenation was therefore investigated over the most selective 2 wt% Pt/SiO_2_ and Pt/SBA-15 catalysts in a stirred batch autoclave under a constant hydrogen pressure between 1 and 10 bar. As anticipated, increasing the hydrogen pressure increased the initial rate and associated TOF of CinnALD hydrogenation (and final conversions) over both supported Pt catalysts (Figure S18). Similar trends have been reported for atmospheric^37^ versus high pressure (48 bar)^50^ CinnALD hydrogenation over carbon nanofiber supported platinum, wherein TOFs rose from ~200 to 828 h^−1^ respectively. While the fumed silica proved slightly more pressure sensitive, the reaction order in *p*H_2_ only ranged from 0.4 to 0.6 between the two supports, close to the 0.5 value expected if the rate-determining step involves the reaction of CinnALD with a single hydrogen adatom originating from the dissociative adsorption of molecular H_2_ (as previously observed for reduced platinum nanoparticles over deoxygenated carbon nanofibers at ambient pressure^50^). The positive order in *p*H_2_ demonstrates that vacant surface sites remain available for dissociative chemisorption of hydrogen over the pressure range explored, with atomic hydrogen participating equally in the two competing pathways for CinnALD hydrogenation to CinnOH or 3-phenyl propionaldehyde, evidenced by the independence of reaction order on product selectivity (see discussion below).

**Figure 8:**
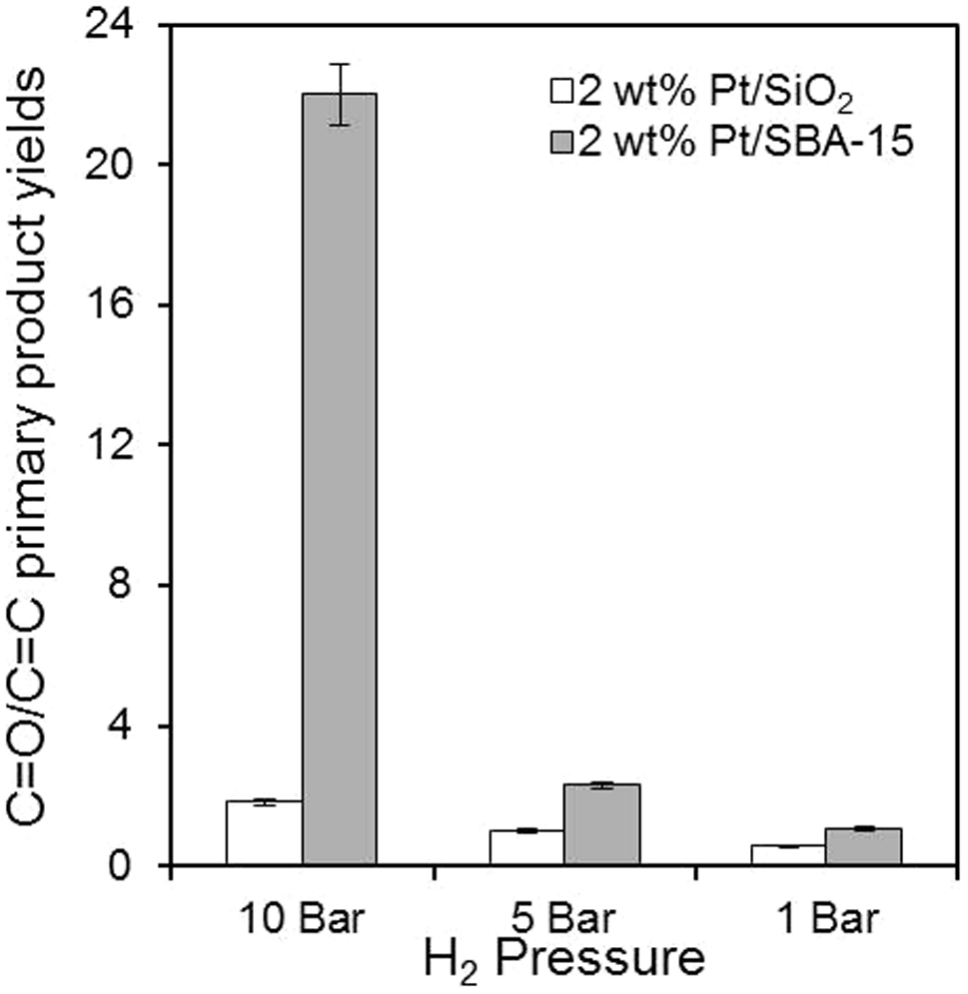
Hydrogen pressure dependence of C = O versus C = C hydrogenation pathways during CinnALD hydrogenation over 2 wt% silica supported Pt catalysts.

The influence of hydrogen pressure upon selectivity (Figure S13) was even more striking than on activity. Figure 8 reveals that CinnALD hydrogenation to CinnOH was favoured over both fumed silica and SBA-15 at higher pressures. Such enhanced selectivity at higher *p*H_2_ was reported for Co-doped Pt nanocrystals, though no explanation was given^20^. For the 2 wt% Pt/SBA-15 catalyst, CinnOH selectivity exceeded 90% at 10 bar, accompanied by trace 3-phenyl propionaldehyde, while for the 2 wt% Pt/SiO_2_ selectivity rose to 56% (predominantly at the expense of ethylbenzene via 3-phenyl propanol hydrogenolysis, Figure S13). This switchover from C = C to C = O hydrogenation with increasing *p*H_2_ is best illustrated by comparing the ratio of CinnOH to 3-phenyl propionaldehyde, which increases 22-fold over the SBA-15 support versus three-fold for the fumed silica, highlighting the greater sensitivity of the mesostructured catalyst to experimental conditions and its superior potential for CinnOH production. Comparison at a common conversion level reveals qualitatively similar trends (Table S3).

The origin of this selectivity enhancement remains unclear, however a number of possibilities occur. High hydrogen pressures may promote Pt nanoparticle restructuring with consequent changes in particle size or exposed facet. In situ XAS measurements by Mistry and co-workers revealed 1 nm platinum clusters underwent a 2D→3D transformation over γ-Al_2_O_3_ with increasing *p*H_2_ 1 to 21 bar at room temperature^58^: specifically, (111) bilayers were proposed to transform into cuboctahedra, which would represent a 70% loss of (111) facets at the expense of (100) facets^59^. Extended Hückel calculations by Delbecq and Sautet suggest that Pt(111) facets favour a di-σ_CO_ CinnALD adsorption mode (and hence C = O hydrogenation), whereas Pt(100) facets favour a co-planar η_4_ mode and hence C = C hydrogenation^44^. For the work of Mistry et al., the high pressure hydrogen-induced switchover from (111) to (100) facets would be predicted to lower selectivity to CinnOH. In order to assess whether such restructuring could occur during high pressure CinnALD hydrogenation, we conducted in situ powder XRD of silica supported 2 wt% Pt catalysts between atmospheric pressure and 7 bar. A small, but systematic fcc platinum lattice expansion (~0.0004 nm ≅ 0.1%) was observed over both supports (Figure S15), being greater for the SBA-15 sample, and which proved largely reversible upon removing hydrogen. Such an expansion is ten times smaller than that observed during the analogous in situ XAS study of Pt/γ-Al_2_O_3_, and the ratio of (111):(200) X-ray reflections was independent of hydrogen pressure, suggesting that hydrogen does not induce significant changes in either nanoparticle shape or size in the present work; this is hardly surprising considering that our 2 wt% catalysts comprise much larger particles of around 15 nm, which thermodynamic calculations predict should exist as stable decahedra^60^,^61^. The magnitude of lattice expansions in Figure S15 are also much less than the 2–4% calculated/experimentally observed for hydride formation^62^,^63^ or hydrogen chemisorption over strained sub-5 nm Pt nanoparticles^64^,^65^,^66^, wherein hydrogen e.g. weakens metal-support interactions, relaxing Pt-Pt distances for smaller particles towards the bulk value. It therefore seems highly unlikely that the selectivity enhancements observed in the present study are attributable to hydrogen-induced restructuring of Pt, but rather a rise in hydrogen surface atom density^58^; concomitant surface crowding destabilising the sterically-demanding η_4_ di-σ_CO_ + π_C = C_ mode thus switching off the C = C hydrogenation pathway. Vergunst et al. proposed a coverage dependent change in CinnALD adsorption mode from flat-lying η_2 _di-σ_CC_ or η_4_ di-σ_CO_ + π_C = C_ to carbonyl end-on adsorption η_2_ di-σ_C = O_ with increasing CinnALD coverage over Pt/C^49^.

### α-Methyl-trans-cinnamaldehyde hydrogenation

**Figure 9:**
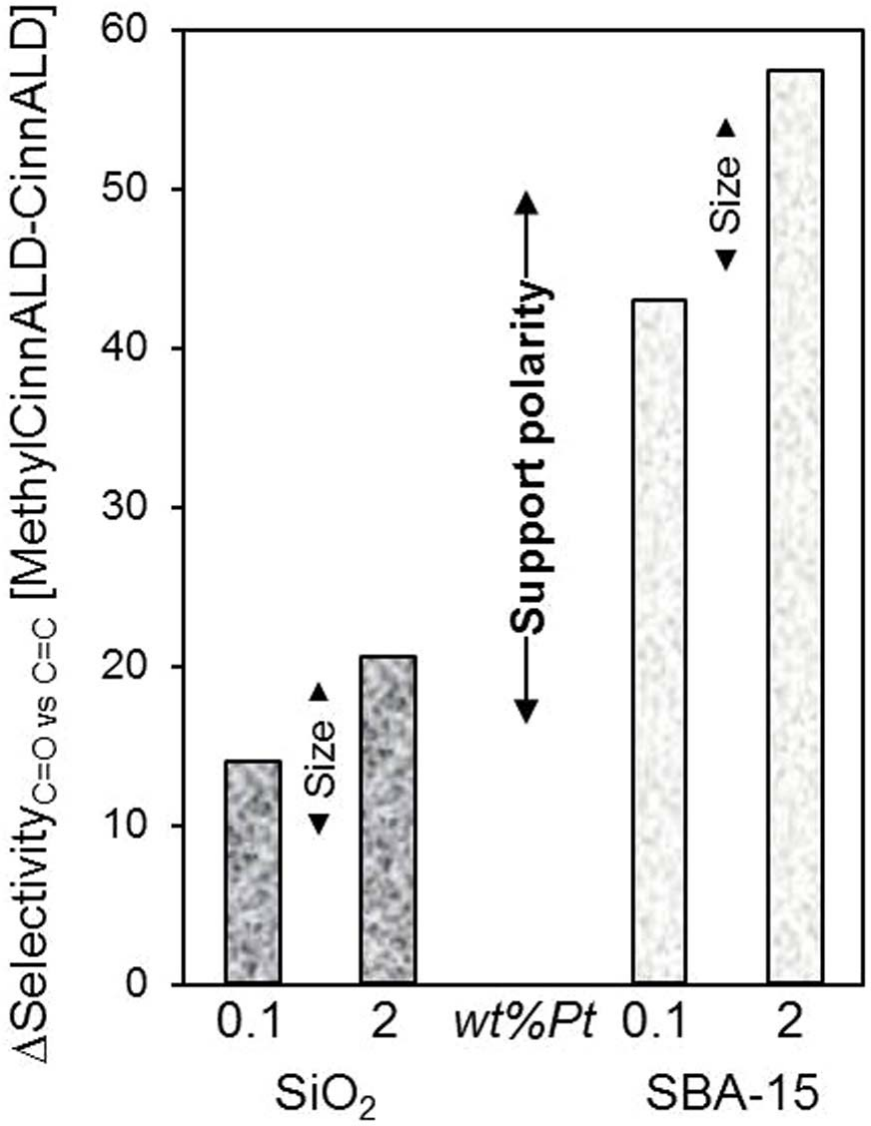
Impact of methyl substitution alpha to the carbonyl in CinnALD upon the rates of C = C versus C = O hydrogenation over small and large Pt nanoparticles supported on fumed or polar mesoporous SBA-15 silicas.

The preceding investigations revealed that destabilisation of C = C relative to C = O adsorption modes of CinnALD over platinum nanoparticles favour its selective hydrogenation to CinnOH. We therefore hypothesised that increasing the steric bulk around the alkene function for a fixed particle size and support polarity, should also hinder di-σ_CC_ or η_4_ di-σ_CO_ + π_C = C_ adsorption and promote the formation of desirable unsaturated alcohols. α-Methyl-*trans*-cinnamaldehyde (2-methyl-3-phenylacrolein) hydrogenation was consequently examined over low and high loading Pt nanoparticles supported on fumed silica and more polar SBA-15. The resulting selectivity to α-methyl-*trans*-cinnamyl alcohol versus 2-methyl-3-phenyl propanol/2-methyl-3-phenyl propionaldehyde was compared with that for CinnALD hydrogenation to CinnOH versus 3-phenyl propan-1-ol/3-phenyl propionaldehyde in Figure 9, i.e. propensity for C = O versus C = C hydrogenation. The results of methyl substitution were striking, significantly favoring C = O hydrogenation to the desired unsaturated alcohol product over C = C hydrogenation to the unsaturated aldehyde/alcohol in all cases by 15–55%. This enhancement was somewhat greater (~10% more) for larger nanoparticles, as anticipated due to increased steric hindrance around the C = C center hindering close approach of the alkene function on extended platinum terraces. However, the impact of silica hydrophilicity was far more dramatic, with selectivity to α-methyl-trans-cinnamyl alcohol enhanced by ~30% over the polar SBA-15 relative to fumed silica, evidencing a strong support effect, with more hydrophobic allylic aldehydes preferentially orientated to favor di-σ_CO_ adsorption on Pt nanoparticles dispersed on polar supports.

## Conclusions

The liquid phase, selective hydrogenation of CinnALD to CinnOH over silica supported Pt nanoparticles strongly depends upon the physicochemical properties of the catalyst and reaction conditions. CinnALD hydrogenation is structure-insensitive with respect to metallic platinum, whereas high selectivity to desired CinnOH product requires large metal ensembles which favor C = O versus C = C hydrogenation. Support polarity also influences product selectivity, with a polar SBA-15 mesoporous silica proving superior to a weakly hydroxylated fumed, low area silica, the former enhancing C = O hydrogenation to the unsaturated alcohol while suppressing its subsequent hydrogenation to 3-phenyl propan-1-ol. In situ ATR-IR surface sensitive spectroscopy implicates a change in CinnALD orientation over the more polar SBA-15 support as the origin of this enhanced selectivity. The generality of this phenomenon was established through the first systematic study of alkyl-substituted benzaldehydes, whose selective carbonyl hydrogenation was similarly promoted over SBA-15 with respect to fumed silica.

Increasing hydrogen pressures between 1→10 bar accelerated CinnALD hydrogenation over both silica supports. However high *p*H_2_ pressures also induced a dramatic switchover in CinnALD reaction pathway from predominantly C = C (1 bar) to >90% C = O hydrogenation (10 bar). In the absence of any apparent change in either platinum oxidation state or morphology, we attribute this significant promotion to the effects of surface crowding upon CinnALD adsorption, with less sterically-demanding η_2_ di-σ_C = O_ binding favored over di-σ_CC_ and η4 di-σ_CO_ + π_C = C_ modes. This hypothesis finds support from experiments on α-methyl-trans-cinnamaldehyde, wherein methylation of the alkene function increases selective hydrogenation of the C = O versus C = C bond with respect to CinnALD, particularly over the polar SBA-15 support for which close approach of the aromatic and methylated alkene functions are disfavored.

Platinum-catalyzed chemoselective hydrogenation of unsaturated aldehydes requires careful tuning of metal particle size/oxidation state *and* support polarity, in concert with high hydrogen pressures in order to achieve high selectivity to the corresponding unsaturated alcohols.

## Methods

### Catalyst synthesis

SBA-15 was synthesised following the method of Stucky and co-workers^34^. Briefly, 10 g Pluronic P123 was dissolved in 75.5 cm^3^ water and 291.5 cm^3^ of 2 M hydrochloric acid under stirring at 35°C. Tetraethylorthosilicate (15.5 cm^3^) was subsequently added and left stirring for 20 h. The resulting gel was aged for 24 h at 80°C without agitation. The solid was filtered, washed with 1000 cm^3^ water, and dried at room temp before calcination at 500°C for 6 h in air (ramp 1°C.min^−1^). The resulting silica exhibited the expected ordered, hexagonal (*p6mm*) arrangements of monodispersed, uniform mesopores.

2 g batches of mesoporous SBA-15 were wetted with 16 cm^3^ of aqueous ammonium tetrachloroplatinate (II) or tetraammine platinum (II) nitrate solutions (precursor concentrations adjusted to achieve nominal Pt loadings spanning 0.05 to 2 wt%). Resulting slurries were stirred for 18 h at room temperature before heating to 50°C. Agitation was ceased after 5 h, and the solids dried for a further 24 h at 50°C to yield a powder. Powder samples were calcined at 500°C for 4 h in air (1°C.min^−1^ ramp rate), prior to reduction at 400°C for 2 h (10°C.min^−1^ ramp rate) under 10 cm^3^.min^−1^ flowing hydrogen. 2 g batches of a mechanically compacted fumed silica (SiO_2_, 200 m^2^g^−1^ S5505 Sigma) were likewise wetted with 16 cm^3^ aqueous ammonium tetrachloroplatinate (II) or tetraammine platinum (II) nitrate solutions (precursor concentrations adjusted to achieve nominal Pt loadings spanning 0.05 to 2 wt%), and the resulting slurries dried, calcined and reduced as above.

### Characterization

Nitrogen porosimetry was undertaken on a Quantachrome Nova 4000e porosimeter and analysed with NovaWin software version 11. Samples were degassed at 120°C for 2 h prior to analysis by nitrogen adsorption at −196°C. Adsorption/desorption isotherms were recorded for all parent and Pt-impregnated silicas. BET surface areas were calculated over the relative pressure range 0.01–0.2. Pore diameters and volumes were calculated by applying the BJH method to desorption isotherms for relative pressures >0.35. Low and wide angle XRD patterns were recorded on a PANalytical X'pertPro diffractometer fitted with an X'celerator detector and Cu K_α _(1.54 Ǻ) source, calibrated against a silicon standard. Low angle patterns were recorded from 2θ = 0.3–8° with a step size of 0.01°, and wide angle patterns from 2θ = 20–90° with a step size of 0.02°. The Scherrer equation was used to calculate volume-averaged Pt crystallite diameters from broadening of the associated metal reflections. In situ XRD was conducted in an Anton Parr XRK900 cell on a Bruker D8 diffractometer employing a Cu K_α _(1.54 Ǻ) source.

XPS was performed on a Kratos Axis HSi X-ray photoelectron spectrometer fitted with a charge neutralizer and magnetic focusing lens, employing Al K_α_ monochromated radiation (1486.7 eV). Spectral fitting was performed using CasaXPS version 2.3.14. Binding energies were corrected to the C 1s peak at 284.6 eV and surface atomic compositions calculated via correction for the appropriate instrument response factors. Pt 4f XP spectra were fitted using a common Gaussian-Lorentzian asymmetric lineshape. Errors were estimated by varying the Shirley background-subtraction procedure and re-calculating component fits. Pt dispersions were measured via CO pulse chemisorption on a Quantachrome ChemBET 3000 system. Samples were outgassed at 150°C under flowing He (20 ml min^−1^) for 1 h, prior to reduction at 150°C under flowing hydrogen (10 ml min^−1^) for 1 h before room temperature analysis; this reduction protocol is milder than that employed during Pt impregnation, and does not induce particle sintering. A CO:Pt_surface_ stoichiometry of 0.68 was assumed, since the formation of a fully saturated monolayer is energetically unfavorable under the measurement conditions employed. DRIFTS measurements were conducted employing a Thermo Scientific Nicolet environmental cell and Smart Collector accessory on a Thermo Scientific Nicolet iS50 FT-IR Spectrometer with MCT detector. Samples were diluted in KBr (1:9) and evacuated at 200°C for 2 h prior to in vacuo spectral acquisition. Attenuated total reflectance IR (ATR-IR) measurements were conducted employing a Pike 20 bounce HATR environmental flow cell and Thermo Scientific Avatar spectrometer with MCT detector and ZnSe ATR crystal. Catalyst films were deposited from aqueous slurries onto the ATR crystal and dried overnight at 40°C in vacuo. A 0.84 M CinnALD in anisole solution was subsequently flowed over the dried catalyst film at 1 ml.min^−1^, and IR spectra recorded as a function of temperature during sample heating at 3°C.min^−1^ from room temperature to 90°C. A liquid-phase vibrational spectrum of CinnALD was calculated for the geometry optimized structure using density functional theory as implemented through Gaussian 03 (Gaussian, Inc., Wallingford CT, 2004) using a 6-311 Gdp basis set and the B3LYP functional in order to aid and visualize spectral assignments.

SEM images were recorded on a Carl Zeiss Evo-40 SEM operating at 10 kV. Samples were supported on carbon tape. Metal loadings were determined using EDX analysis at 25 kV with a maximum current of 25 nA and working distance of 9 mm. High resolution (S)TEM images were recorded on an FEI Tecnai F20 field emission gun TEM operated at 200 kV equipped with a Gatan Orius SC600A CCD camera. Samples were prepared for TEM by dispersion in ethanol and drop-casting onto a copper grid coated with a continuous carbon support film (Agar Scientific Ltd). Images were analyzed in ImageJ 1.41. The Gaussian width of Pt nanoparticle size distributions from which mean values are reported in Figures 1, 2 was only ~±0.5 nm, which does not represent a significant variance considering the overall size range spanned of 1.9–16 nm nor interferes with the size dependent selectivity and activity reported in this work.

### Cinnamaldehyde hydrogenation

Catalyst testing was performed using a Radleys Starfish parallel reactor on a 10 cm^3^ scale at 90°C. 100 mg of catalyst were added to reaction mixtures containing 8.4 mmol of aldehyde substrate in 10 cm^3^ anisole solvent, and 0.1 cm^3^ internal standard (mesitylene) at 90°C under 700 rpm stirring and bubbling H_2_ (1 bar, 5 cm^3^.min^−1^) which ensured the absence of external mass-transport limitations. Reactivity of the 2 wt% Pt/SBA-15 catalyst was also independent of silica particle size, confirming facile in-pore mass-transport (Table S4). The absolute Pt content varied between 0.26 μmol (0.05 wt% catalysts) and 10.8 μmol (for the highest loading 2.10 wt%), corresponding to substrate:catalyst ratios ranging from 3.28 × 10^4^ (0.05 wt%) down to 6.92 × 10^2^ (2.1 wt%). Reactions were sampled periodically for kinetic profiling by off-line gas chromatography using a Varian 3800GC with 8400 autosampler fitted with a VF-5ms Factor Four column (30 m × 0.25 mm × 0.25 μm). For CinnALD, catalytic hydrogenation of the likely reaction intermediates 3-phenyl propan-1-ol, 3-phenyl propionaldehyde, 3-phenyl propanoic acid, cinnamic acid and CinnOH was also measured for the lowest and highest Pt loadings (0.05 and 2.1 wt%) on both silicas under identical conditions to those employed for CinnALD hydrogenation. The role of hydrogen pressure was investigated keeping other reaction conditions (temperature, internal standard and substrate:catalyst ratio) identical, within a stirred Parr 5513 100 ml stainless steel autoclave between 1 and 10 bar H_2_ pressure; activity and selectivity were assessed through periodic sampling via a dip-tube. Control experiments verified negligible substrate conversion in the absence of either H_2_ or platinum catalyst, while hot filtration tests evidenced no detectable metal leaching, confirming the heterogeneous nature of the observed reactions. Quoted activities and selectivities are the mean of duplicate or triplicate reactions with errors ±2%; mass balances >95% in all cases. Conversion, selectivity, yield and TOF were defined as below:
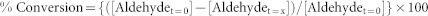

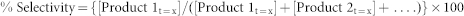

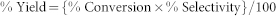

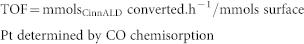


## Author Contributions

A.F.L. and K.W. conceived the research programme. L.J.D. synthesized all catalysts, conducted porosimetry, D.R.I.F.T.S., A.T.R.-I.R., X.R.D. and X.P.S. characterization, and performed all hydrogenation reactions. N.S.H. undertook HRTEM measurements and assisted with analysis. C.M.A.P. assisted with the design and analysis of DRIFTS experiments. M.A.I. assisted with XPS measurements and analysis. The manuscript was written through contributions of all authors.

## Supplementary Material

Supplementary Information Supporting information
