# Supplementary material for: Selectivity control in Pt-catalyzed cinnamaldehyde hydrogenation
Source: Sci Rep. 2015 Mar 24;5:9425. doi: 10.1038/srep09425 (PMC4371104; doi:10.1038/srep09425)
Supplement: Supplementary Information — Supporting information [file srep09425-s1.pdf]

Selectivity control in Pt-catalysed cinnamaldehyde hydrogenation

Lee J. Durndell,[†] Christopher M.A. Parlett,[†] Nicole S. Hondow,[¶] Mark A. Isaacs,[†] Karen Wilson[†] and Adam F. Lee[†*]

[†]European Bioenergy Research Institute, Aston University, Aston Triangle, Birmingham, B4 7ET, UK
[¶]Institute for Materials Research, School of Process, Environmental and Materials Engineering, University of Leeds, Leeds, LS2 9JT, UK

Structural properties of parent silica supports

Low angle powder XRD (**Figure S1**) and high resolution TEM (**Figure S2**) evidence successful synthesis of the parent SBA-15 support, with textural properties from porosimetry entirely consistent with the literature (**Table S1**).

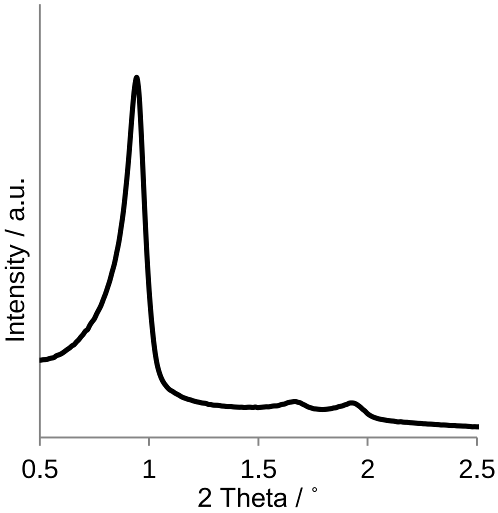

Figure S1. Low angle XRD pattern of parent SBA-15 support highlighting reflections associated with hexagonal close packed arrangement of mesopores.

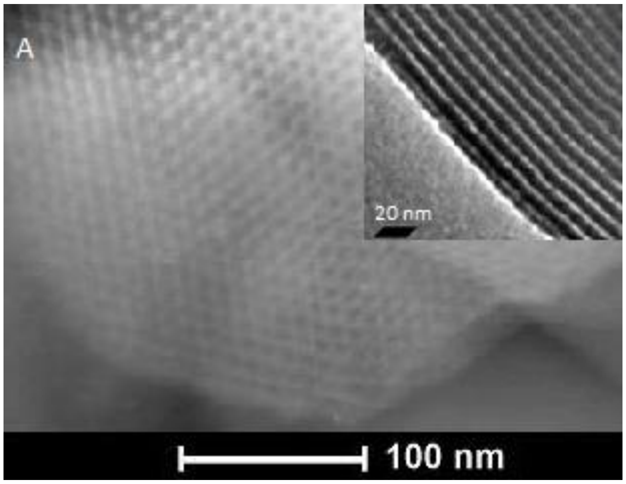

Figure S2. Dark-field high resolution TEM micrograph of parent SBA-15 support highlighting hexagonally close-packed mesopores (inset shows bright-field micrograph highlighting parallel mesopore channels).

Table S1. Textural properties of parent supports determined from powder XRD and N_2 porosimetry.

Sample	Surface area / $m^2\ g^{-1}$ [(a)]	Mesopore surface area / $m^2.g^{-1}$	Micropore surface area / $m^2.g^{-1}$	Mean mesopore diameter / nm[(b)]	Lattice Parameter / nm[(c)]	Pore Separation / nm[(c)]
Fumed SiO_2 (Sigma - S5505)	208	171	37	31.3	n/a	n/a
SBA-15	932	491	441	5.8	9.0	10.4

[a]N_2 BET, [b]BJH desorption isotherm, [c]Low angle XRD via Bragg's law

Structural properties of Pt impregnated silica supports

Low angle powder XRD (**Figure S3**) and porosimetry confirmed the textural properties of the parent fumed and SBA-15 silicas were retained following impregnation with Pt (**Table S2**).

Table S2. Physicochemical properties of supported Pt nanoparticles

Support	Precursor	Pt loading / wt%[(a)]	Pt dispersion / %[(b)]	Pt particle size / nm[(c)]	PtO_2 content / %[(d)]	Surface area / $m^2\ g^{-1}$[(e)]	Micropore surface area / $m^2\ g^{-1}$[(e)]	Mean mesopore diameter / nm[(f)]
SiO_2	Chloride	2.10	10.0	15.6 (16.6)	7.7	176.16	35.16	n/a
SiO_2	Chloride	0.96	14.7	9.7 (9.5)	10.1	188.25	37.25	n/a
SiO_2	Chloride	0.54	20.0	7.7 (8.4)	16.2	192.50	37.21	n/a
SiO_2	Chloride	0.09	44.4	3.4 (N/A)	23.2	194.36	36.09	n/a
SiO_2	Chloride	0.05	52.4	3.0 (N/A)	26.7	189.93	35.93	n/a
SiO_2	Nitrate	1.48	9.4	15.7 (14.1)	6.7	202.38	36.01	n/a
SiO_2	Nitrate	0.92	14.4	9.8 (8.9)	8.8	205.79	37.19	n/a
SiO_2	Nitrate	0.31	31.1	4.2 (3.9)	12.6	192.00	37.41	n/a
SiO_2	Nitrate	0.09	51.3	3.1 (N/A)	31.7	205.82	36.99	n/a
SiO_2	Nitrate	0.05	56.0	2.8 (N/A)	34.8	202.55	36.25	n/a
SBA-15	Chloride	2.01	14.9	10.3 (11.4)	13.8	530.69	163.21	5.8
SBA-15	Chloride	1.00	18.3	7.9 (9.0)	14.8	584.45	201.04	5.8
SBA-15	Chloride	0.52	31.8	3.8 (5.4)	18.1	679.22	259.41	5.8
SBA-15	Chloride	0.11	70.9	2.3 (N/A)	33.8	742.19	330.18	5.8
SBA-15	Chloride	0.06	82.5	1.9 (N/A)	36.0	764.20	346.37	5.8
SBA-15	Nitrate	1.42	19.9	7.8 (8.2)	10.3	573.84	197.25	5.8
SBA-15	Nitrate	0.34	42.3	3.5 (4.1)	19.3	691.46	271.86	5.8
SBA-15	Nitrate	0.11	76.8	2.1 (N/A)	28.4	735.36	327.44	5.8

[a]SEM/EDX; [b]from CO chemisorption assuming a CO:$Pt_{surface}$ stoichiometry of 0.68[1, 2]; [c]From CO chemisorption with parenthesis values from XRD via Scherrer analysis; [d]XPS; [e]N_2 BET; [f]BJH desorption isotherm

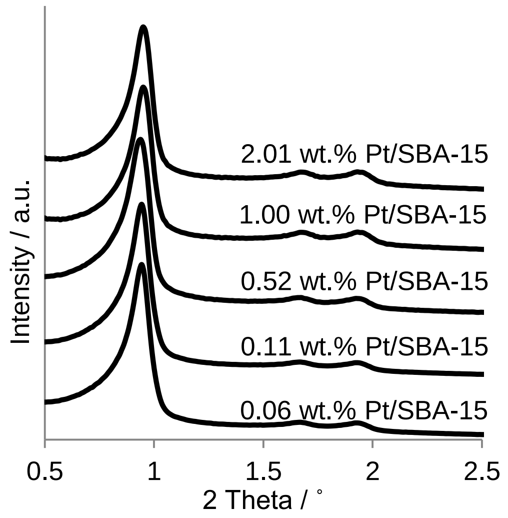

Figure S3. Low angle XRD patterns of Pt impregnated SBA-15 samples evidencing ordered mesoporosity of parent SBA-15. Offset for clarity.

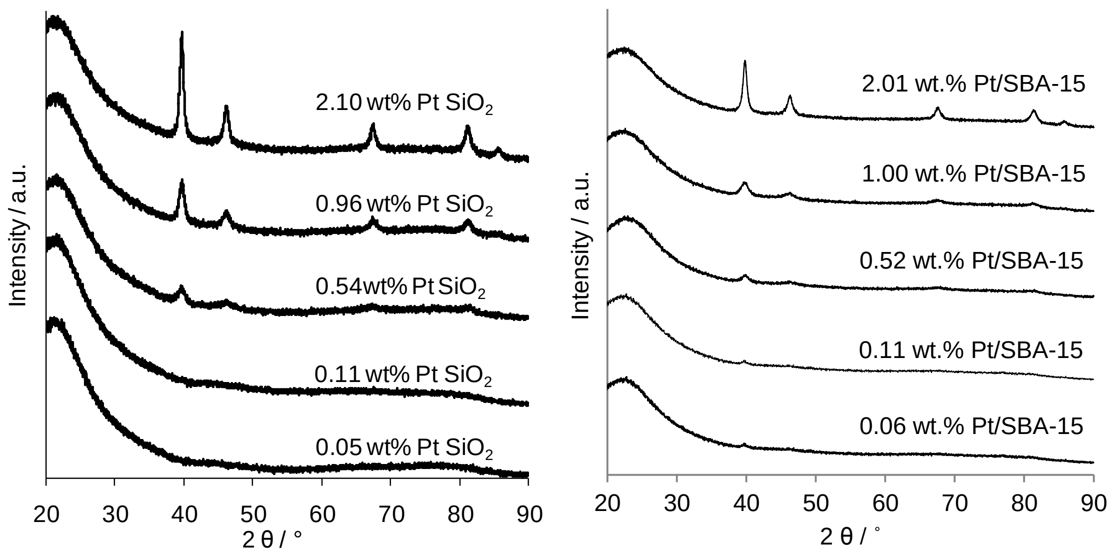

Figure S4. Wide angle XRD patterns of Pt impregnated SBA-15 and fumed silica showing the emergence of fcc Pd crystallites at high loadings. Offset for clarity.

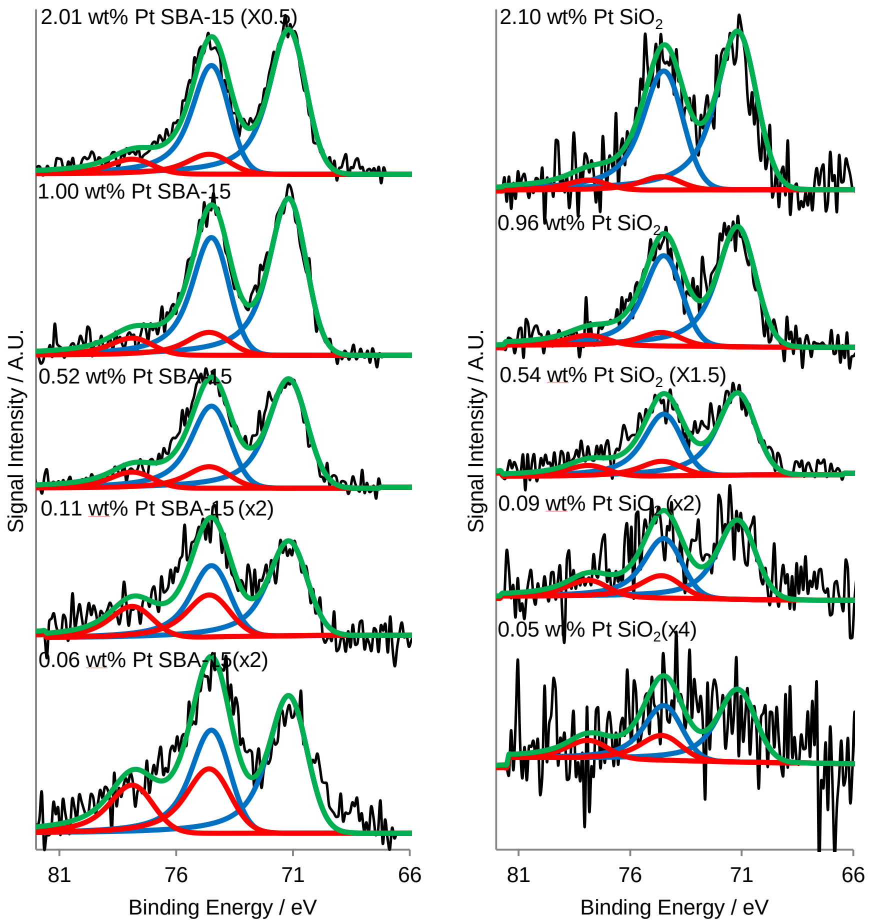

Figure S5. Pt 4f XP spectra of Pt impregnated SBA-15 (left) and fumed silica (right). Contributions due to $Pt^{(0)}$ (blue) and PtO_2 (red) fitted components are shown, revealing the emergence of oxide features at low loadings. The solid green line represents the fitted envelope. Offset for clarity.

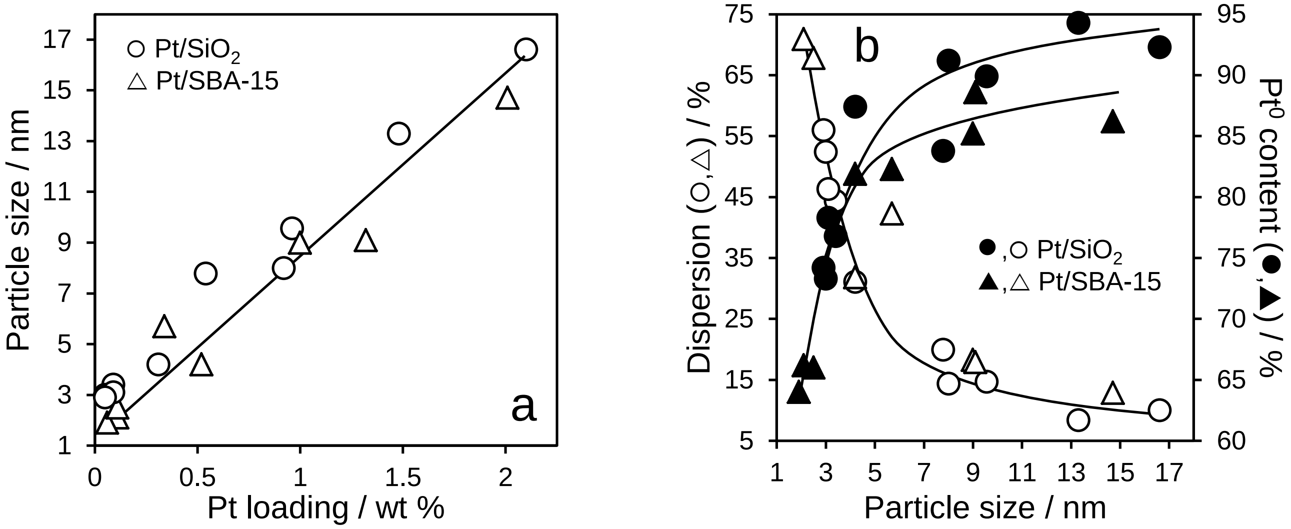

Figure S6. Nanoparticle (a) size dependence on bulk Pt loading, and (b) dispersion and surface oxidation state dependence on size over fumed and mesoporous SBA-15 silicas.

Catalytic hydrogenation of cinnamaldehyde (CinnALD) over Pt impregnated silica supports

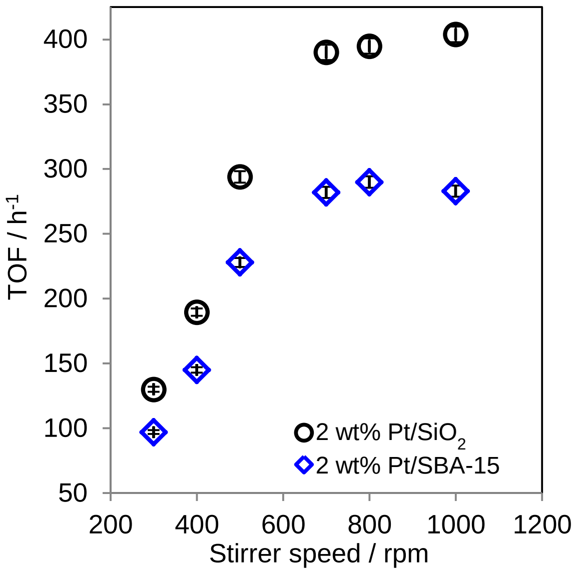

Figure S7. Influence of stirring rate on TOFs for cinnamaldehyde hydrogenation over 2 wt% Pt/SiO_2 and 2 wt% Pt/SBA-15 catalysts, showing elimination of external mass-transport limitations above 800 rpm.

NOTE: All reaction data reported in **Figures S8-14** were obtained at a stirrer rate of 700 rpm to ensure the absence of external mass-transport limitations.

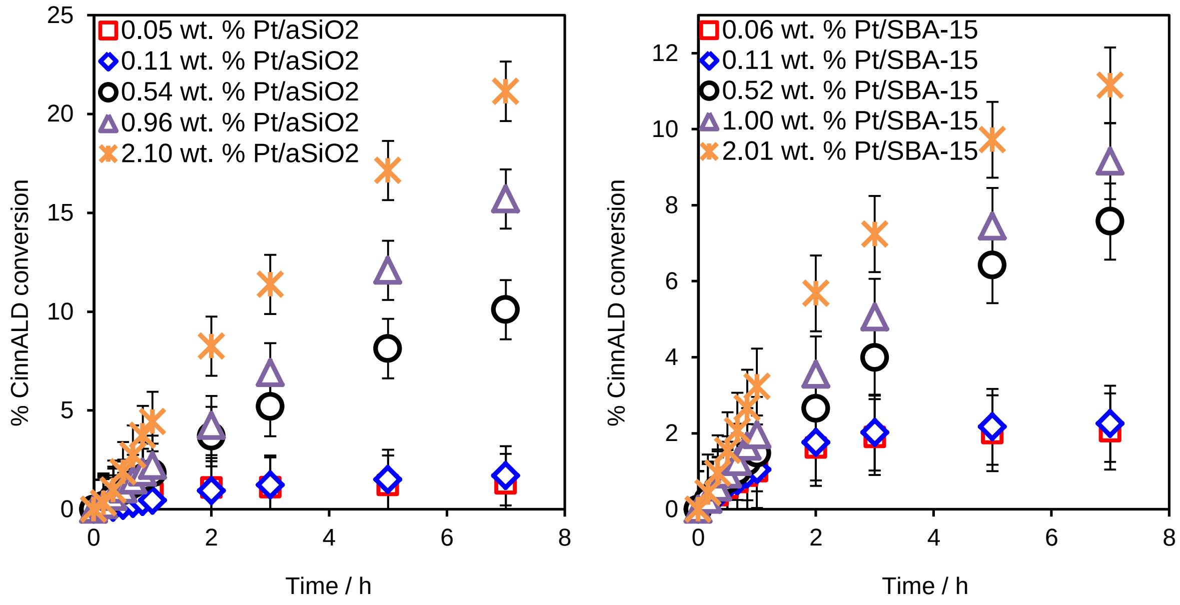

Figure S8. Cinnamaldehyde hydrogenation reaction profiles for fumed silica (left) and SBA-15 (right) series under ambient conditions.

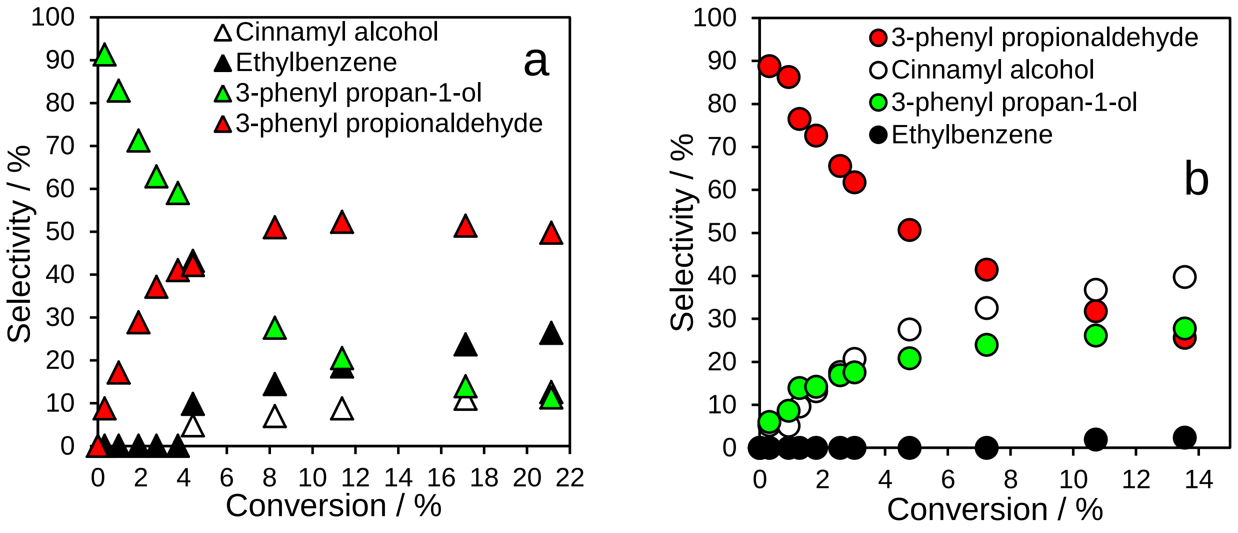

Figure S9. Evolution of product selectivity as a function of cinnamaldehyde conversion over 2 wt% (left) Pt/SiO_2 and (right) Pt/SBA-15 catalysts.

Note: during the early stages of reaction over 2 wt% Pt/SiO_2 the principal product is 3-phenyl propan-1-ol, a secondary product arising from hydrogenation of either cinnamyl alcohol or 3-phenyl propionaldehyde. The absence of primary products, particularly cinnamyl alcohol, during this early stage of reaction can however be rationalised by considering that the rate of cinnamyl alcohol hydrogenation (and hence removal from the reaction mixture) is >40 times faster than its initial

rate of formation. This would result in a very low concentration of cinnamyl alcohol and high concentration of 3-phenyl propan-1-ol secondary product according to the kinetic scheme below (from **Figure 4**):

$$\text{Cinnamaldehyde} \xrightarrow[\textit{slow}]{\textit{21}} \text{Cinnamyl alcohol} \xrightarrow[\textit{v. fast}]{\textit{883}} \text{3-Phenyl propan-1-ol}$$

The apparently paradoxical accumulation of primary product at higher conversions may then be understood in terms of deactivation of the second hydrogenation step. In contrast over 2 wt% Pt/SBA-15 the rates of cinnamyl alcohol formation (74 $mmol.h^{-1}.g_{Pt}^{-1}$) and its removal via hydrogenation to 3-phenyl propan-1-ol (30 $mmol.h^{-1}.g_{Pt}^{-1}$) are reversed.

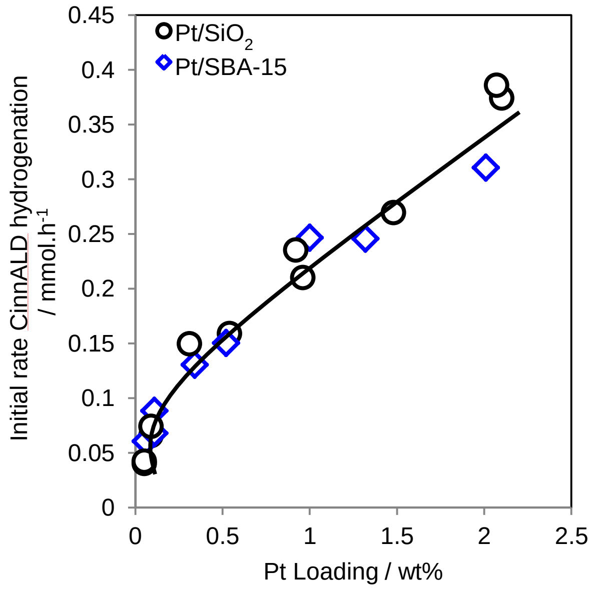

Figure S10. Initial rates as a function of Pt loading.

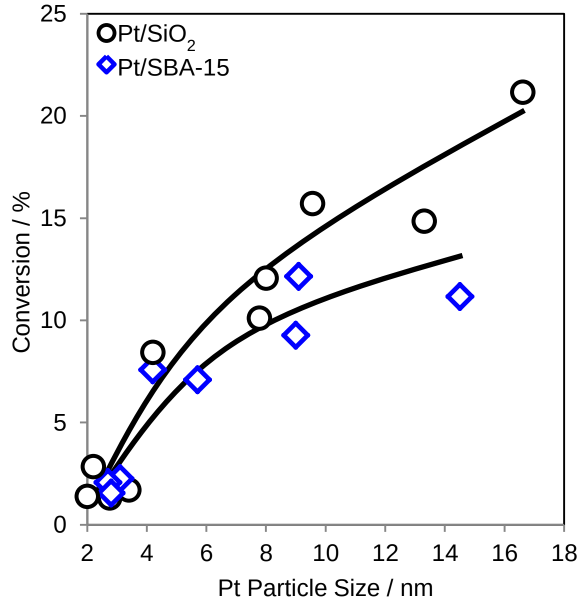

Figure S11. Effect of Pt particle size on cinnamaldehyde conversion after 7 h on fumed and SBA-15 silica supports.

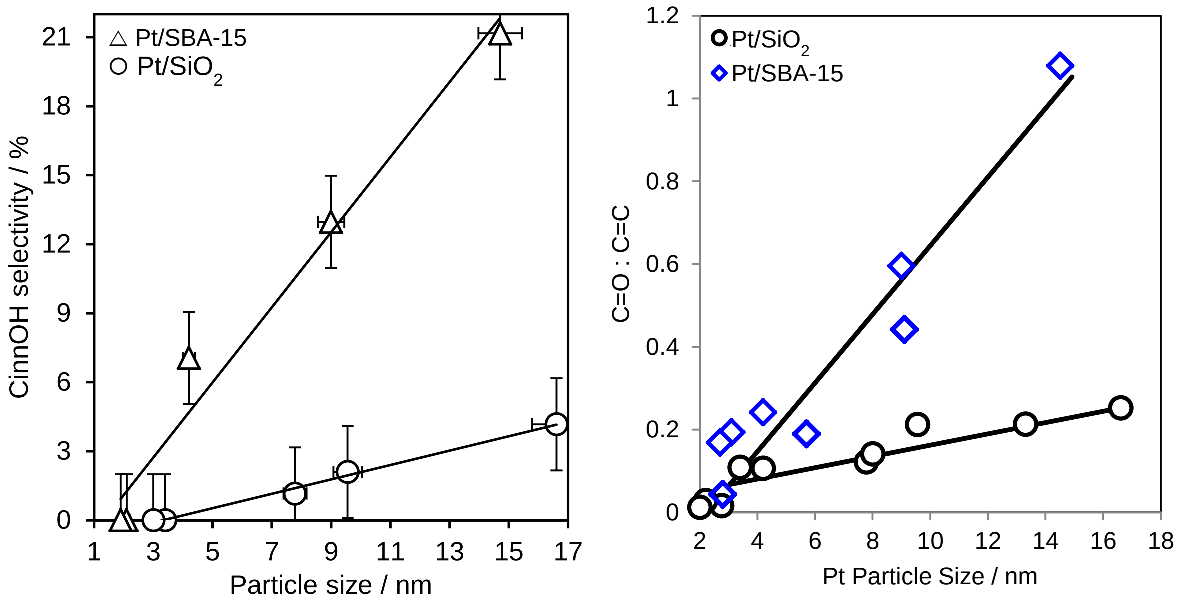

Figure S12. (left) cinnamyl alcohol selectivity after 1 h cinnamaldehyde hydrogenation over silica supported platinum catalysts at 1 bar as a function of particle size; (right) C=O versus C=C hydrogenation ratio as a function of Pt particle size at 1 bar.

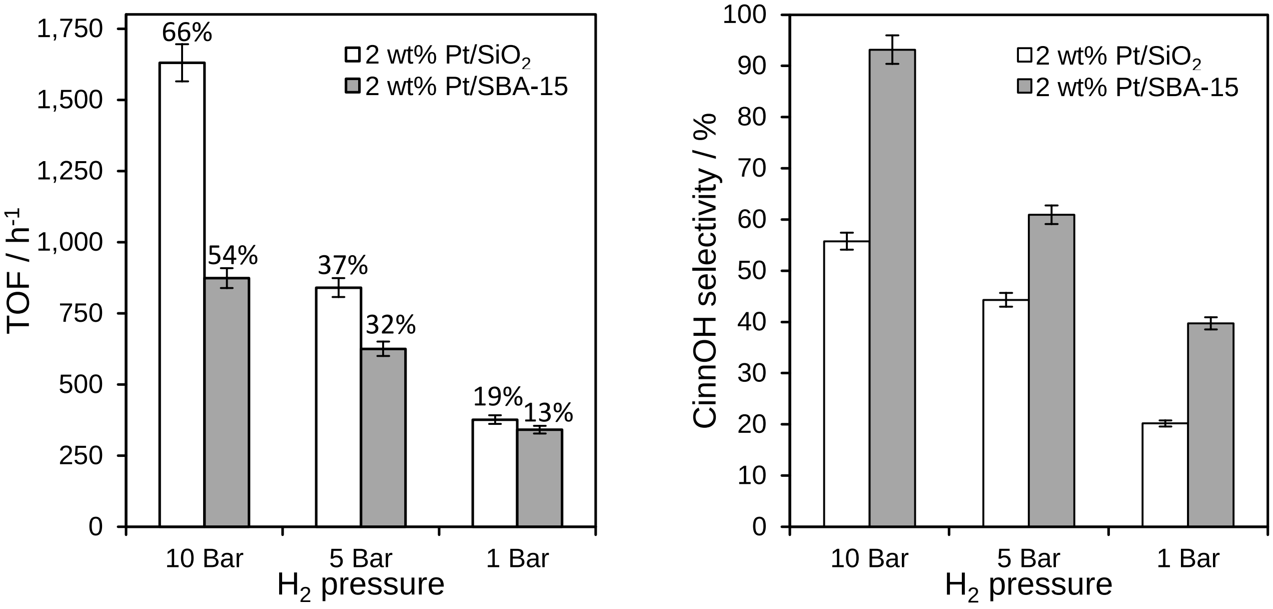

Figure S13. Hydrogen pressure dependence of CinnALD hydrogenation over 2 wt% silica supported Pt catalysts: (left) TOFs and inset 7 h conversions; and (right) selectivity to CinnOH after 7 h reaction.

Table S3. Selectivity of 2 wt% Pt/silica catalysts at constant CinnALD conversion

			Product Selectivity		
**Sample**	**Pressure**	**Conv. %**	**CinnOH**	**3-PhenALD**	**Ethylbenzene**
**2 wt% Pt/SiO_2**	**1**	**13.5**	9.9	51.0	21.3
	**5**		27.5	46.7	14.6
	**10**		38.0	49.8	4.1
**2 wt% Pt/SBA-15**	**1**		39.7	25.6	2.4
	**5**		53.3	21.1	1.9
	**10**		61.0	16.8	0.0

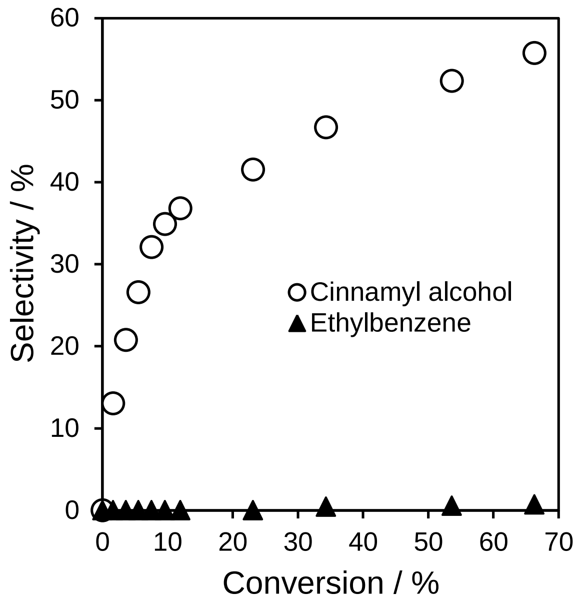

Figure S14. Cinnamyl alcohol selectivity as a function of conversion over 2 wt% Pt/SiO_2 at 10 bar.

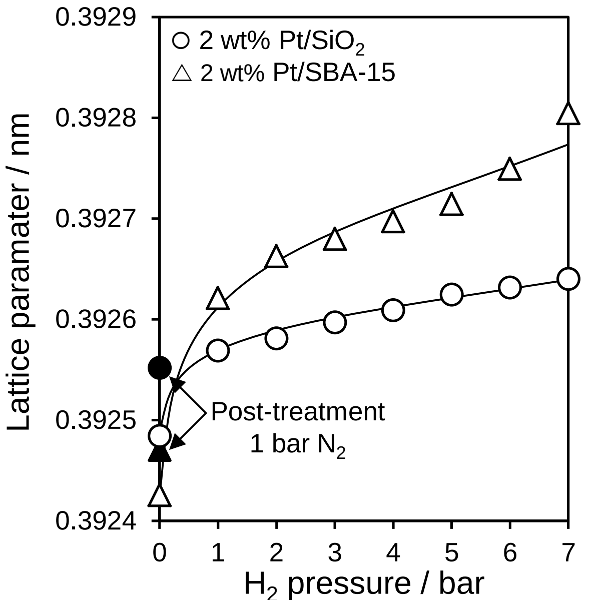

Figure S15. Hydrogen pressure dependence of fcc platinum lattice parameter of 2 wt% silica supported Pt catalysts at 90 °C.

Table S4. Influence of crystallite size on cinnamaldehyde hydrogenation for 2 wt% Pt/SBA-15 evidencing absence of internal (in-pore) mass transport limitations

	**Selectivity at 7 h**			
**Product**	**Unsieved**	**0.6 mm**	**0.25 mm**	**0.15 mm**
CinnOH	13.6	16.2	16.2	15.3
3PhenOH	11.3	14.7	12.2	13.3
Ethylbenzene	25.1	23.8	22.6	24.6
3PhenALD	49.8	44.0	47.7	45.6
CinnACID	0.0	1.2	1.3	1.2
3PhenACID	0.0	0.0	0.0	0.0
Ester	1.27	0.0	0.0	0.0
**Initial rate mmol.g_{cat}^{-1}.h^{-1}**	0.42	0.44	0.44	0.46
